# Supplementary material for: Resolving the full spectrum of human genome variation using Linked-Reads
Source: Genome Res. 2019 Apr;29(4):635–45. doi: 10.1101/gr.234443.118 (PMC6442396; doi:10.1101/gr.234443.118)
Supplement: Supplemental Material [file supp_gr.234443.118_Supplemental_longranger-2.2.zip › longranger-2.2.tar.bz2!/lr2.2-public/lib/stan/cmdstan/stan/doc/api/html/agrad_2fwd_2matrix_2divide_8hpp_source.html]

Stan: src/stan/agrad/fwd/matrix/divide.hpp Source File


|  |  |
| --- | --- |
|  | Stan  2.5.0  probability, sampling & optimization |


- Main Page
- Related Pages
- Namespaces
- Classes
- Files

- File List
- File Members

All Classes Namespaces Files Functions Variables Typedefs Enumerations Enumerator Friends Macros Pages

- src
- stan
- agrad
- fwd
- matrix

divide.hpp

Go to the documentation of this file.

1 #ifndef STAN\_\_AGRAD\_\_FWD\_\_MATRIX\_\_DIVIDE\_HPP

2 #define STAN\_\_AGRAD\_\_FWD\_\_MATRIX\_\_DIVIDE\_HPP

3

4 #include <vector>

5 #include <stan/math/matrix/Eigen.hpp>

6 #include <stan/agrad/fwd.hpp>

7 #include <stan/agrad/fwd/operators/operator\_division.hpp>

8 #include <stan/agrad/fwd/matrix/to\_fvar.hpp>

9 #include <stan/agrad/fwd/matrix/typedefs.hpp>

10

11 namespace stan {

12  namespace agrad {

13

14  template <typename T1, typename T2>

15  inline

16  typename stan::return\_type<T1,T2>::type

17  divide(const T1& v, const T2& c) {

18  return v / c;

19  }

20

21  template <typename T, int R, int C>

22  inline Eigen::Matrix<fvar<T>,R,C>

23  divide(const Eigen::Matrix<fvar<T>, R,C>& v, const fvar<T>& c) {

24  Eigen::Matrix<fvar<T>,R,C> res(v.rows(),v.cols());

25  for(int i = 0; i < v.rows(); i++) {

26  for(int j = 0; j < v.cols(); j++)

27  res(i,j) = v(i,j) / c;

28  }

29  return res;

30  }

31

32  template <typename T, int R, int C>

33  inline Eigen::Matrix<fvar<T>,R,C>

34  divide(const Eigen::Matrix<fvar<T>, R,C>& v, const double c) {

35  Eigen::Matrix<fvar<T>,R,C>

36  res(v.rows(),v.cols());

37  for(int i = 0; i < v.rows(); i++) {

38  for(int j = 0; j < v.cols(); j++)

39  res(i,j) = v(i,j) / c;

40  }

41  return res;

42  }

43

44  template <typename T, int R, int C>

45  inline Eigen::Matrix<fvar<T>,R,C>

46  divide(const Eigen::Matrix<double, R,C>& v, const fvar<T>& c) {

47  Eigen::Matrix<fvar<T>,R,C>

48  res(v.rows(),v.cols());

49  for(int i = 0; i < v.rows(); i++) {

50  for(int j = 0; j < v.cols(); j++)

51  res(i,j) = v(i,j) / c;

52  }

53  return res;

54  }

55

56  template <typename T, int R, int C>

57  inline Eigen::Matrix<fvar<T>,R,C>

58  operator/(const Eigen::Matrix<fvar<T>, R,C>& v, const fvar<T>& c) {

59  return divide(v,c);

60  }

61

62  template <typename T, int R, int C>

63  inline Eigen::Matrix<fvar<T>,R,C>

64  operator/(const Eigen::Matrix<fvar<T>, R,C>& v, const double c) {

65  return divide(v,c);

66  }

67

68  template <typename T, int R, int C>

69  inline Eigen::Matrix<fvar<T>,R,C>

70  operator/(const Eigen::Matrix<double,R,C>& v, const fvar<T>& c) {

71  return divide(v,c);

72  }

73  }

74 }

75 #endif

stan::agrad::operator/

Eigen::Matrix< fvar< T >, R, C > operator/(const Eigen::Matrix< fvar< T >, R, C > &v, const fvar< T > &c)

**Definition:** divide.hpp:58

stan::agrad::fvar

**Definition:** fvar.hpp:13

fwd.hpp

operator\_division.hpp

typedefs.hpp

stan::return\_type::type

boost::math::tools::promote\_args< typename scalar\_type< T1 >::type, typename scalar\_type< T2 >::type, typename scalar\_type< T3 >::type, typename scalar\_type< T4 >::type, typename scalar\_type< T5 >::type, typename scalar\_type< T6 >::type >::type type

**Definition:** traits.hpp:406

Eigen.hpp

to\_fvar.hpp

stan::agrad::divide

stan::return\_type< T1, T2 >::type divide(const T1 &v, const T2 &c)

**Definition:** divide.hpp:17


---

[ Stan Home Page ]

*© 2011–2014,
Stan Development Team.*
